# 

**Published:** 1913-07-12

**Authors:** 


### Personalia.

MR. C. F. SHULER, fifth assistant medical officer at Banstead Mental Hospital, has resigned his appointment.

MR. W. H. ECROYD has been appointed a member of the Bexley Mental Hospital Sub-Committee of the London County Council.

MR. GEORGE DEW has been appointed a member of the Banstead Mental Hospital Sub-Committee of the London County Council.

DR. F. S. PALMER, physician to the West End Hospital, has been elected president of the West London Medical Society for the ensuing year.

CAPTAIN KIMMITT has been elected honorary secretary of the King Edward Memorial Hospital, Ealing, in succession to the late Mr. William Hedges. Captain Kimmitt has been acting for some time as assistant honorary secretary. Major Goldney has resigned the office of organising secretary, and, at his request, his services have been regarded as honorary.

### Gifts and Bequests.

THE late Duchess of Northumberland presented an *x*-ray apparatus to the Alnwick Infirmary.

THE Chelsea Hospital for Women has received £100 from the Goldsmiths' Company towards its rebuilding fund.

MR. FRANCIS RECKITT has given £20,000 to the Newland Orphan Homes of the Port of Hull Society and Sailors' Orphan Homes.

MR. GEORGE EMIL ADOLPHUS REISS, of Henley-on-Thames, has left £2,500 to his sons with the request, but without creating any obligation, that they will give £500 to the Eccles and Patricroft Hospital.

MRS. ELIZA ANN STANLEY, of South Norwood, has left Ordinary Shares in Messrs. W. F. Stanley and Co. (Limited) to servants for life, with remainders to the Royal Merchant Seamen's Orphanage, the Homes for the Aged Poor, South Norwood, and to the Metropolitan and City Police Orphanage.

MISS MARY LOUISA VOILE, of Cheltenham, has left £1,000 to the Victoria Nursing Home, £1,000 to the Charity Organisation Society, £2,000 to the General Hospital, all of Cheltenham; and £500 each to the Delancey Fever Hospital and the Girls' Orphanage. These are duty free, and the residue is to be divided between the London Cancer Hospital and the United Kingdom Beneficent Association.

